# How life course socioeconomic conditions shape multimorbidity in old age – a scoping review

**DOI:** 10.1093/eurpub/ckac129.502

**Published:** 2022-10-25

**Authors:** C Wagner, C Carmeli, A Chiolero, S Cullati

**Affiliations:** 1 Population Health Laboratory, University of Fribourg, Fribourg, Switzerland; 2 Institute of Primary Health Care, University of Bern, Bern, Switzerland; 3 School of Population and Global Health, McGill University, Montreal, Canada; 4 Department of Readaptation and Geriatrics, University of Geneva, Geneva, Switzerland

## Abstract

Multimorbidity is the co-existence of two or more chronic conditions in the same individual and is highly prevalent in ageing populations. Inequity in multimorbidity in old age could have socioeconomic roots that can be traced across the life course. It is, however, unclear if adverse socioeconomic conditions (SEC) at different periods of the life course predict the occurrence of multimorbidity in later life. We reviewed, therefore, studies assessing the association between life course SEC, measured at min. two time points, and later-life multimorbidity. We identified four studies (25,209 participants) with the first measure of SEC in childhood. In these four studies, childhood SEC was associated with multimorbidity in old age, and the associations were partially or fully attenuated upon adjustment for later-life SEC. We identified five studies (91,236 participants) with the first measure of SEC in young adulthood, and the associations with multimorbidity in old age as well as the effects of adjustment for later-life SEC differed from one study to the other. In conclusion, SEC in early life could have an effect on multimorbidity, attenuated at least partly by SEC in adulthood. Our results suggest that interventions and health promotion aiming to reduce the risk of multimorbidity in old age should target early-life socioeconomic conditions.

